# Clinical Outcomes of Semi-Occlusive Dressing for Allen Type III Fingertip Injuries

**DOI:** 10.3390/jcm15197372

**Published:** 2026-09-23

**Authors:** Jinsik Park, In Cheul Choi, Chanmo Jeong, Young Seok Lee, Chang-Hun Lee

**Affiliations:** 1 Department of Orthopaedic Surgery, Hanyang University Guri Hospital, Guri 11923, Republic of Korea; gizumyong@gmail.com (J.P.); grampus2000@gmail.com (Y.S.L.); 2 Department of Orthopaedic Surgery, Korea University Anam Hospital, Korea University College of Medicine, Seoul 02841, Republic of Korea; indolldr@korea.ac.kr; 3 Department of Orthopaedic Surgery, Hanyang University College of Medicine, Seoul 04763, Republic of Korea; jcm11043@gmail.com

**Keywords:** fingertip amputation, conservative treatment, semi-occlusive dressing, regeneration, wound healing, recovery of function

## Abstract

**Background/Objectives:** Local flap coverage is still widely regarded as the treatment of choice for Allen type III fingertip injuries, and outcome data for conservative treatment remain limited. We evaluated the outcomes of semi-occlusive dressing for Allen type III fingertip injuries with bone exposure. **Methods:** This retrospective single-centre case series included 15 patients with 17 digits sustaining Allen type III fingertip injuries with exposed bone treated with semi-occlusive dressing. Allen type I or II injuries, isolated pulp defects, infection-related tissue loss and incomplete follow-up were excluded. Finger and thumb motion were assessed as total active motion and interphalangeal joint motion, respectively, as a percentage of the contralateral digit. Sensation was assessed using two-point discrimination, Semmes–Weinstein monofilament testing and a subjective contralateral percentage scale. **Results:** All 17 digits healed at a mean of 36 days (range 21–60), with a mean follow-up of 25 months (range 12–63). No additional surgery or major complications occurred. Nail deformity developed in four of the 17 digits (24%). Among the 14 fingers, total active motion averaged 95% of the contralateral side (range 80–100) and ten regained full motion; the three thumbs regained mean interphalangeal joint motion of 80% (range 50–100). The mean two-point discrimination was 6 mm, the median Semmes–Weinstein value was 2.83 and the mean subjective sensation was 84%. **Conclusions:** In Allen type III fingertip injuries with bone exposure, semi-occlusive dressing achieved reliable healing with satisfactory functional and sensory recovery and may be a useful alternative or adjunct to surgical reconstruction.

## 1. Introduction

Fingertip injuries are common traumatic hand injuries that require appropriate management to restore function, sensation and appearance [1,2]. Traditional locoregional flap techniques, including V–Y advancement, homodigital island, thenar and cross-finger flaps, have been used to provide soft tissue coverage and preserve digital length [3]. However, flap procedures have limitations. Many require advanced surgical skills and have a steep learning curve [4], and reconstruction often necessitates operative facilities, anaesthesia, prolonged operative time and postoperative immobilization, increasing the treatment burden and cost while potentially delaying functional recovery [5]. Donor site morbidity, including scarring, sensory disturbance and deformities of adjacent digits, is a concern [6].

To overcome these limitations, conservative strategies have been extensively explored, among which semi-occlusive dressing has been applied as a modality [7,8,9]. Occlusive or semi-occlusive wound management can achieve reliable healing with satisfactory functional and sensory outcomes, even when bone is exposed [9,10], with lower rates of joint stiffness and reduced treatment burden compared with flap reconstruction [11,12].

Recent evidence has strengthened the case for conservative management. A systematic review of 15 studies and 611 fingertip amputations reported complete healing in 97.4% of digits at a mean of 30 days, full range of motion in 93% of patients and a need for surgical intervention in 1.5% [13]. That review, however, confined its conclusion to amputations in which bone was not exposed, noted that the inclusion of Allen type I injuries could obscure the performance of the technique in more severe injuries, and recommended that future studies analyze outcomes according to the Allen type [13]. Once the amputation reaches Allen type III, local flap coverage is still widely regarded as the treatment of choice [14], and series confined to this level remain few: such injuries have mostly been reported as subgroups within broader cohorts [14,15] or pooled with less severe injuries and with fingertip necrosis [16]. We therefore restricted this study to Allen type III fingertip injuries with bone exposure and report injury characteristics, healing time, functional and sensory recovery and complications for each digit so that outcomes can be related to the severity of the individual injury.

## 2. Materials and Methods

### 2.1. Study Design and Patients

This retrospective case series reviewed patients treated with semi-occlusive dressing for fingertip injuries at a single centre between April 2020 and June 2026. Clinical data, including demographic characteristics, injury patterns, treatment details and clinical outcomes, were obtained from electronic medical records.

Patients were eligible if they had an Allen type III fingertip injury with bone exposure, treated with semi-occlusive dressing as the primary wound management modality, either alone or in combination with a surgical procedure, and had records documenting the wound course until complete epithelialization. Allen type I and II injuries, isolated pulp defects without bone loss, injuries with soft tissue loss due to infection and records with insufficient documentation were excluded. Patients who could not complete functional and sensory assessment, for example, because of death from unrelated disease or loss to follow-up before evaluation, were excluded to ensure complete outcome data among the analyzed digits. The unit of analysis was the injured digit rather than the patient. When a patient sustained injuries to multiple digits, only the digit or digits with an Allen type III fingertip injury with bone exposure treated with semi-occlusive dressing were included; concomitantly injured digits treated by other means were excluded.

Twenty-seven patients treated with semi-occlusive dressing during the study period were screened. Twelve were excluded: four had an injury that did not meet the criteria for an Allen type III injury, one had a soft tissue defect without bone exposure, two had soft tissue loss due to infection, two had an amputation proximal to the fingertip that was managed by stump revision, and three died of unrelated disease before the functional and sensory assessment. Fifteen patients with 17 injured digits were therefore analyzed.

### 2.2. Semi-Occlusive Dressing Technique

After the initial assessment, the wound was irrigated, any necrotic tissue was debrided, and the digit was soaked in a 1:1 mixture of povidone–iodine and normal saline for 10–15 min. A semi-occlusive film dressing (Ioban 2 Antimicrobial Incise Drape; 3M Health Care, St. Paul, MN, USA) was then wrapped circumferentially around the digit and sealed beyond the fingertip, enclosing the wound in a closed moist chamber (Figure 1). The dressing was changed weekly at the outpatient clinic until complete epithelialization was achieved without wound discharge.

When a surgical procedure was performed before the dressing, it was done only for a defined indication, such as a grossly contaminated wound, arterial (pumping) bleeding requiring hemostasis, or the presence of a repairable skin or nail bed flap, rather than routinely. In all patients who underwent surgery, the procedure comprised a uniform set of components: irrigation of contaminants, debridement of necrotic tissue, hemostasis of active bleeding and primary repair of the skin and nail bed when feasible. Semi-occlusive dressing was then applied in the same manner as in patients treated without surgery.

### 2.3. Outcome Assessment

Healing time was defined as the interval from the date of injury to complete epithelialization without wound discharge, confirmed at the outpatient clinic.

Injury characteristics were recorded for each digit. The orientation of the defect was classified as transverse, volar-oblique, dorso-oblique or lateral oblique; defects that were irregular and could not be assigned to any of these orientations were recorded as not classifiable. Nail bed injury was recorded as the deepest layer involved (confined to the sterile matrix, extending to the germinal matrix, or extending proximal to the nail fold), as the proportion of the total nail bed area lost, and as whether primary nail bed repair had been performed at the initial procedure. These variables were determined from the operative records, clinical notes and wound photographs obtained at presentation.

The outcomes included wound healing, functional recovery and complications. For the fingers, range of motion was assessed as total active motion and expressed as a percentage of the corresponding contralateral finger, with full recovery defined as a value equal to that of the contralateral side (100%). Because total active motion is not applicable to the thumb, thumb motion was assessed as interphalangeal joint motion and expressed as a percentage of the contralateral thumb. Sensory recovery was assessed using static two-point discrimination, Semmes–Weinstein monofilament testing and a subjective scale expressed as a percentage of the contralateral digit, obtained during clinical examination or patient interview at follow-up. Two-point discrimination was measured with a hand evaluation caliper and monofilament testing with a five-filament set comprising the markings 2.83, 3.61, 4.31, 4.56 and 6.65 (Baseline 12-0117 and 12-1740; Fabrication Enterprises, White Plains, NY, USA). Nail deformities were recorded when present and described by type. All functional and sensory assessments were performed in person at the final follow-up visit, at a mean of 25 months after injury (range 12–63 months).

### 2.4. Statistical Analysis

Descriptive statistics were used. Continuous variables are presented as means with ranges, ordinal variables as medians with ranges, and categorical variables as frequencies and percentages. Semmes–Weinstein monofilament results were treated as ordinal, because the filament index markings form a discrete and unequally spaced logarithmic scale; they are summarized as medians and reported at the markings of the filament set. The two treatment subgroups—digits treated with the dressing alone and digits treated after a surgical procedure—were compared using two-sided exact tests: an exact permutation Mann–Whitney U test using midranks for continuous and ordinal variables, Fisher’s exact test for binary variables, and the Fisher–Freeman–Halton exact test for the overall distributions of injury mechanism and defect orientation. Age and sex were analyzed per patient and all other variables per digit. Associations between injury characteristics and nail deformity were examined with Fisher’s exact test and the Mann–Whitney U test, and the relationship between follow-up duration and sensory outcome was examined with the Spearman rank correlation coefficient. No adjustment was made for multiple comparisons, and all *p*-values are regarded as exploratory. Because the subgroup comparison was not a primary aim of the study and the subgroups were small, the absence of a statistically significant difference was not interpreted as evidence of equivalence. Analyses were performed with Python 3.11 and SciPy 1.11.

## 3. Results

Fifteen patients with 17 injured digits met the inclusion criteria and had complete outcome data (Table 1). The cohort comprised eleven men and four women with a mean age of 58 years (range 39–79). The injured digits were the thumb in three, index finger in three, middle finger in one, ring finger in five and small finger in five. Two patients sustained multiple digit injuries. By the inclusion criterion, all injuries had bone exposure. The most common mechanisms were machinery-related injury (seven patients) and crushing (five patients), followed by cutting (two) and other mechanisms (one).

The defect was transverse in eight digits, dorso-oblique in five and lateral oblique in one; in the remaining three digits, all of which resulted from a crushing or multiple-slice mechanism, the defect was irregular and could not be assigned to any of these orientations. No digit had a volar-oblique defect. Injury was confined to the sterile matrix in 13 digits and extended to the germinal matrix in four, and the mean nail bed loss was 61% of the nail bed area (range 10–100).

Semi-occlusive dressing was the primary treatment modality for all patients. Seven patients (eight digits) were treated with the dressing alone and eight patients (nine digits) underwent the dressing after a surgical procedure; primary nail bed repair was performed in six of the latter digits.

Complete wound healing was achieved in all 17 digits at a mean of 36 days (range 21–60), and the mean follow-up was 25 months (range 12–63). No digit required additional surgery after the initial treatment, and no major complication such as wound necrosis or non-healing occurred. For range of motion, the 14 fingers had a mean total active motion of 95% of the contralateral side (range 80–100), of which ten regained full motion, and the three thumbs regained a mean of 80% (range 50–100) of contralateral interphalangeal joint motion (Table 2). For sensory recovery, the mean two-point discrimination was 6 mm (range 3–10), the median Semmes–Weinstein value was 2.83 (range 2.83–4.31) and the mean subjective sensation was 84% (range 20–100) of the contralateral digit. Injury characteristics, treatment and outcomes for each digit are given in Table 3. The clinical course of a representative digit treated with the dressing alone is shown in Figure 2, and a further example is shown in Figure 3.

A comparison of the two treatment subgroups is presented in Table 4. The subgroups differed in the pattern of injury: all five crushing injuries and all three irregular defects occurred in digits treated after a surgical procedure (mechanism, *p* = 0.03), and healing took longer in that subgroup (43 versus 28 days, *p* = 0.04), although this difference was no longer significant when each patient contributed a single averaged value (*p* = 0.08). The Semmes–Weinstein value was higher after surgery (median 3.61 versus 2.83, *p* = 0.02). No difference was detected between the subgroups in nail bed loss, germinal matrix involvement, total active motion, nail deformity, two-point discrimination or subjective sensation.

Sensory outcome was not associated with the duration of follow-up (two-point discrimination, Spearman ρ = 0.44, *p* = 0.08; subjective sensation, ρ = −0.11, *p* = 0.68). The apparent trend for two-point discrimination was driven by the single digit with both the longest follow-up and the poorest sensory recovery; after excluding this digit, the association was weaker (ρ = 0.32, *p* = 0.23).

## 4. Discussion

In this cohort of Allen type III fingertip injuries with bone exposure, semi-occlusive dressing achieved complete wound healing in every digit at a mean of approximately 36 days, without secondary surgery. Functional recovery was generally favourable, with most fingers regaining full or near-full motion and satisfactory sensation, and no major complication occurred. Digits treated with the dressing alone and those treated after a surgical procedure differed in the pattern of injury but reached comparable functional outcomes.

These findings are consistent with those of previous studies. In a comparative study, Pastor et al. reported a 100% healing rate, favourable pain and sensory outcomes and shorter work absence with semi-occlusive dressing, whereas 28% of surgically treated patients failed to heal because of graft necrosis [12]. Likewise, Söderberg et al. [10] and Hoigné et al. [11] reported reliable soft tissue regeneration after conservative dressing of fingertip amputations with exposed bone. The healing interval in our series closely corresponds to these reports. These favourable outcomes may be related to the biological benefits of the moist wound environment created by the dressing, which promotes granulation, epithelial migration and healing by secondary intention [9]. By avoiding flap reconstruction, donor site morbidity is prevented and the stiffness associated with prolonged immobilization may be reduced [1,3,6]. These advantages are relevant in patients with extensive soft tissue loss for whom conventional reconstruction carries a considerable surgical burden [7,8].

Fewer data are available for injuries at the Allen type III level. Three series have reported semi-occlusive dressing in injuries of comparable severity. Quadlbauer et al. treated nine Allen type III and four type IV injuries within a cohort of 77 and found no residual restriction of finger motion, with normal light touch sensation in 77% of these 13 digits [14]. Randazzo et al. compared occlusive dressing with local flap coverage in 56 fingers with exposed bone, of which seven were Ishikawa zone 3, and reported complete healing at a mean of five weeks, better two-point discrimination than after flap coverage and a lower rate of proximal interphalangeal joint stiffness [15]. Dimitrova-Chakarova et al. treated 50 patients with semi-occlusive dressing combined with a surgical procedure and found that outcomes in Allen type III and IV injuries were comparable to those in type I and II injuries [16]. Our healing interval of 36 days and the preservation of finger motion fall within the range described by these series.

The present series adds to these reports in three respects. First, the cohort was restricted to Allen type III injuries with bone exposure rather than analyzed within a broader cohort spanning several injury grades, so the outcomes reported here relate to a single, clearly defined injury level. Second, motion and sensation were assessed with the same contralateral-referenced metrics in every digit, and all assessments were made in person at final follow-up. Third, injury characteristics and outcomes are reported for each digit (Table 3), which allows the reader to relate individual outcomes to the severity of the corresponding injury.

The decision to perform a surgical procedure before the dressing followed defined indications—gross contamination, arterial bleeding requiring hemostasis, or the presence of a repairable skin or nail bed flap—rather than an overall judgement of injury severity. A digit with a large or proximal defect was not operated on if the wound was clean, was not bleeding actively and offered no tissue worth repairing.

When the two subgroups were nevertheless compared retrospectively, several differences emerged (Table 4). All five crushing injuries and all three irregular defects occurred among the digits treated after a surgical procedure, healing in that subgroup took approximately two weeks longer, and the Semmes–Weinstein value was higher, indicating poorer light touch sensation. One explanation is that the operative indications themselves are met more often in high-energy injuries, since contamination and active bleeding accompany crushing and machinery injuries more frequently than clean cuts. Combining an initial surgical procedure with semi-occlusive dressing in this way has previously been reported to be compatible with good fingertip formation [16]. The subgroups did not differ in the extent of nail bed loss or in germinal matrix involvement, and total active motion, the rate of full recovery of motion, nail deformity and subjective sensation were similar.

This comparison was not a primary aim of the study. The subgroups are small and the analysis is exploratory; the differences observed should be interpreted with caution, and the absence of a detected difference does not establish equivalence.

The motion outcomes were favourable for most digits. Among the fingers, total active motion averaged 95% of the contralateral side and reached full recovery in approximately three-quarters, indicating that healing by secondary intention under a semi-occlusive dressing, which avoids prolonged immobilization, generally preserves joint motion. Thumb motion assessed at the interphalangeal joint averaged 80% of the contralateral side. The few digits with reduced motion were those with the most extensive initial injury, suggesting that residual stiffness reflects the severity of the injury itself rather than the dressing.

Sensory recovery after fingertip injury depends principally on the extent of pulp loss, which the Allen classification does not capture because it is based on the plane of amputation. In this series the defect orientation provides a partial substitute for that information. Eight defects were transverse, five dorso-oblique and one lateral oblique, and three were irregular; no digit sustained a volar-oblique defect, the pattern associated with the greatest loss of pulp tissue. The favourable sensory values observed here should therefore be read in that context: they describe a cohort in which the pulp was comparatively spared, and they should not be taken to indicate that semi-occlusive dressing restores sensation after extensive pulp loss. Defect orientation is only an indirect indicator of the extent of pulp loss, and prospective studies that grade pulp loss independently of the amputation plane would allow the sensory potential of this approach to be defined more precisely.

Nail deformity is a recognized concern after treatment of fingertip injuries at or proximal to the nail bed level, and it developed in four of the 17 digits in our series: two hook nails and two dystrophic nails (Figure 4). This overall rate is broadly comparable with previous conservative series: in a randomized trial, Bensa et al. found that semi-occlusive dressing achieved healing and sensory recovery similar to surgical reconstruction but was associated with a higher rate of nail deformity, particularly in more proximal injuries [17], and comparable proportions have been reported for conservative and surgical treatment of fingertip amputations with bone exposure [10,11]. Randazzo et al. similarly observed a hook nail in every Ishikawa zone 3 amputation in their series, although the two classifications are not directly interchangeable [15].

The digit-level data suggest that the extent of nail bed loss, rather than its depth or its repair, distinguished the digits that developed a deformity. All four deformities occurred among the six digits with a nail bed loss of 80% or more, and none among the 11 digits with a lesser loss; the mean nail bed loss was 88% in the affected digits and 53% in the remainder. Neither germinal matrix involvement nor primary nail bed repair separated the two groups. A similar direction has been reported by Dimitrova-Chakarova et al., who observed that nail growth disturbance became more frequent the shorter the remaining distal phalanx, approaching every digit in Allen type IV injuries [16]. With four events our observation cannot be treated as a predictive rule, and it requires confirmation in a larger series. It is nevertheless consistent with the view that the nail outcome is determined mainly by the amount of nail bed irrecoverably lost at the time of injury rather than by the method of subsequent wound management.

Neither patient with a hook nail requested or required secondary correction. Neither reported pain, recurrent infection or catching of the nail, and functional impairment was minimal; the deformity was accepted on cosmetic grounds. Correction of an established hook nail requires nail bed revision, usually with bone or matrix grafting, and is generally reserved for symptomatic deformity, which was not the case in either patient.

This study has several limitations. The retrospective design carries a risk of selection bias. Patients who could not complete the functional and sensory assessment were excluded, and if their outcomes were poorer than those of the patients assessed, the results reported here would be overestimates. The follow-up interval also varied widely. Two patients had two injured digits each, so not all 17 digits were statistically independent; repeating the subgroup comparison with one value per patient removed the difference in healing time. The subgroup comparison was based on small numbers and does not support conclusions about either treatment. Finally, no digit in this series had a volar-oblique defect, so the sensory outcomes reported here may not extend to injuries with greater pulp loss. The absence of a control group and the small sample size preclude any conclusion about the superiority of this approach over flap reconstruction.

## 5. Conclusions

In this retrospective case series of Allen type III fingertip injuries with bone exposure, semi-occlusive dressing achieved complete wound healing in all digits, with satisfactory functional and sensory recovery and without secondary surgery or major complications. These findings suggest that, in appropriately selected patients with this injury pattern, semi-occlusive dressing can be considered as part of the management of Allen type III injuries, either alone or following an initial surgical procedure. Prospective comparative studies are required to define its role relative to flap reconstruction.

## Figures and Tables

**Figure 1:**
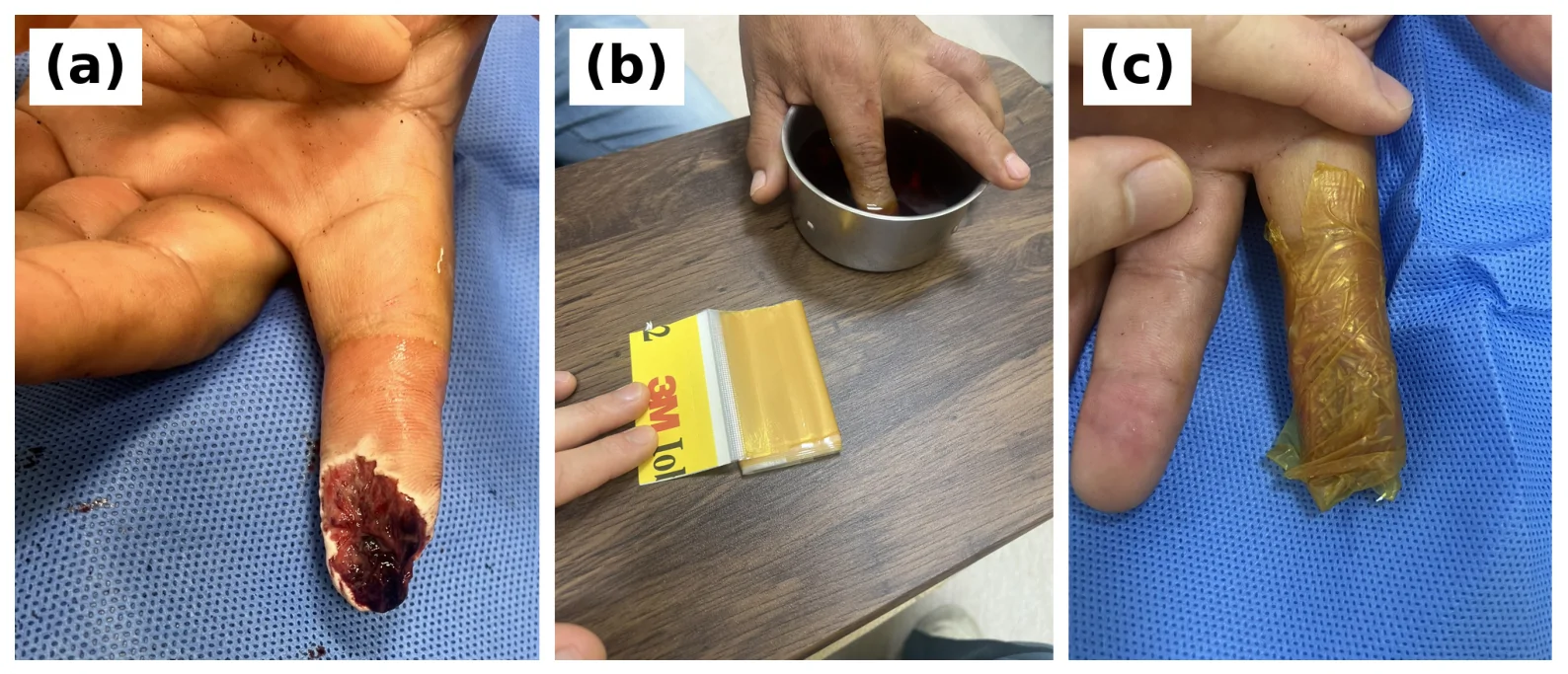
Application of the semi-occlusive dressing. (**a**) Allen type III fingertip injury with exposed bone after initial irrigation and debridement. (**b**) The digit is soaked in a 1:1 mixture of povidone–iodine and normal saline, and the semi-occlusive film (Ioban 2 Antimicrobial Incise Drape; 3M Health Care, St. Paul, MN, USA) is prepared. (**c**) The film is wrapped circumferentially around the digit and sealed beyond the fingertip, enclosing the wound in a closed moist chamber.

**Figure 2:**
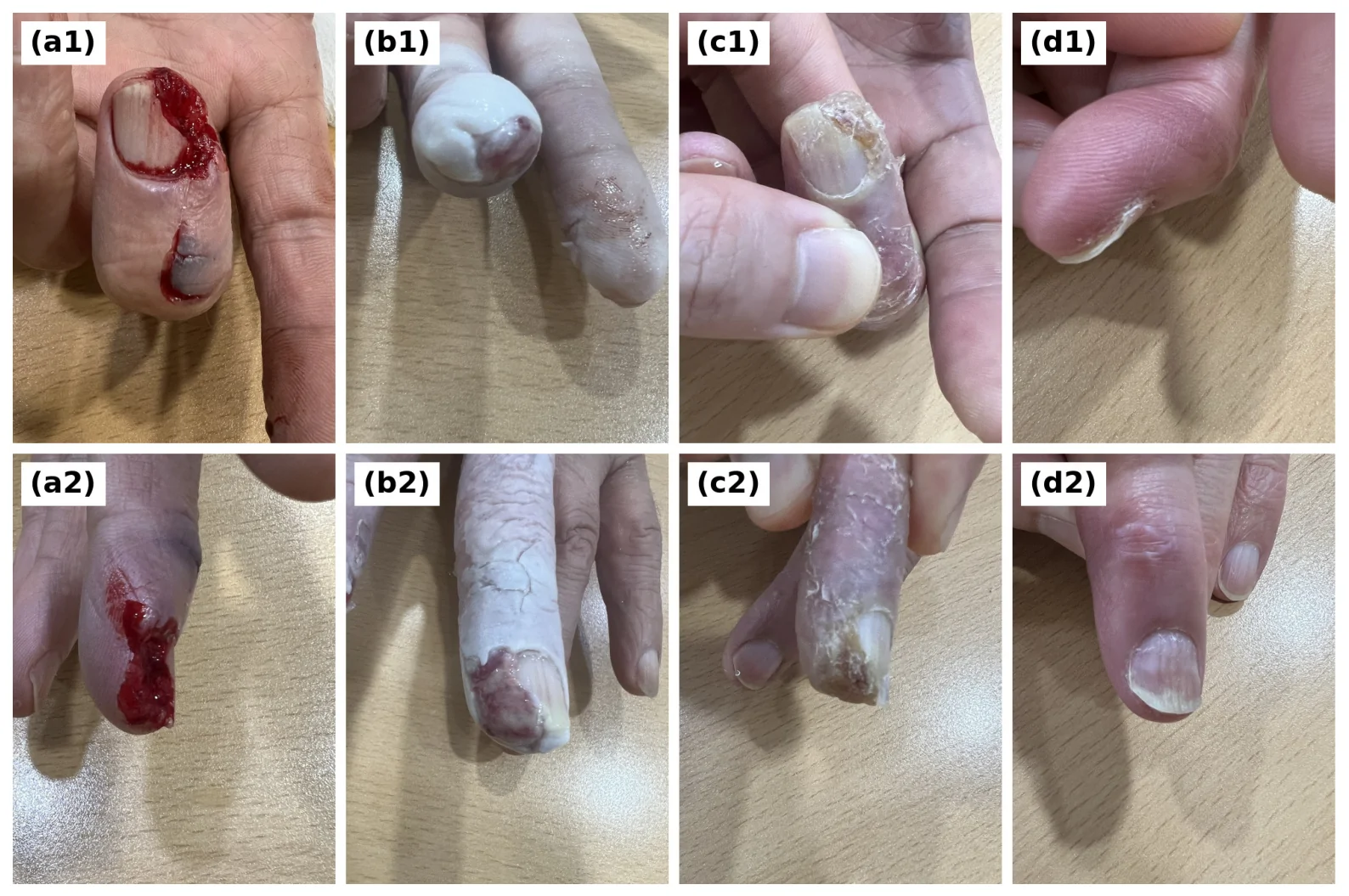
Clinical course of an Allen type III fingertip injury of the left middle finger (digit no. 5) treated with semi-occlusive dressing alone. (**a1**,**a2**) Injury day: lateral oblique amputation with exposed distal phalanx and loss of approximately 30% of the nail bed, limited to the sterile matrix. (**b1**,**b2**) Day 10: granulation tissue filling the defect, with the characteristic maceration of the perilesional skin produced by the moist environment. (**c1**,**c2**) Day 30: complete epithelialization, corresponding to the healing time recorded for this digit. (**d1**,**d2**) Day 90: mature contour with a regenerated nail plate and restoration of the fingerprint ridges.

**Figure 3:**
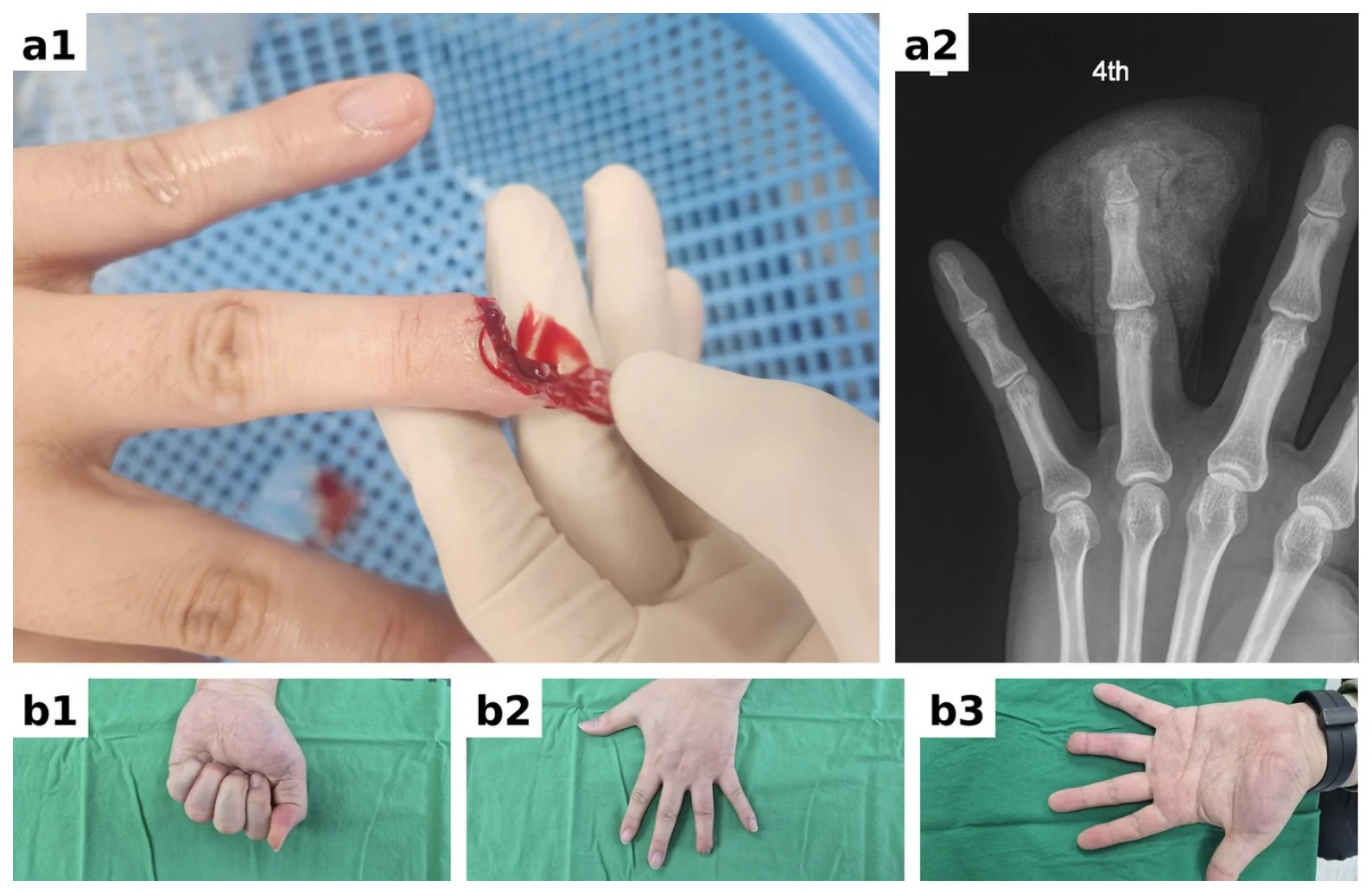
Allen type III ring finger injury (digit no. 16) treated with semi-occlusive dressing alone. (**a1**,**a2**) Injury day: clinical photograph (**a1**) and radiograph (**a2**). (**b1**–**b3**) Final follow-up: flexion (**b1**), dorsal view (**b2**) and extension (**b3**).

**Figure 4:**
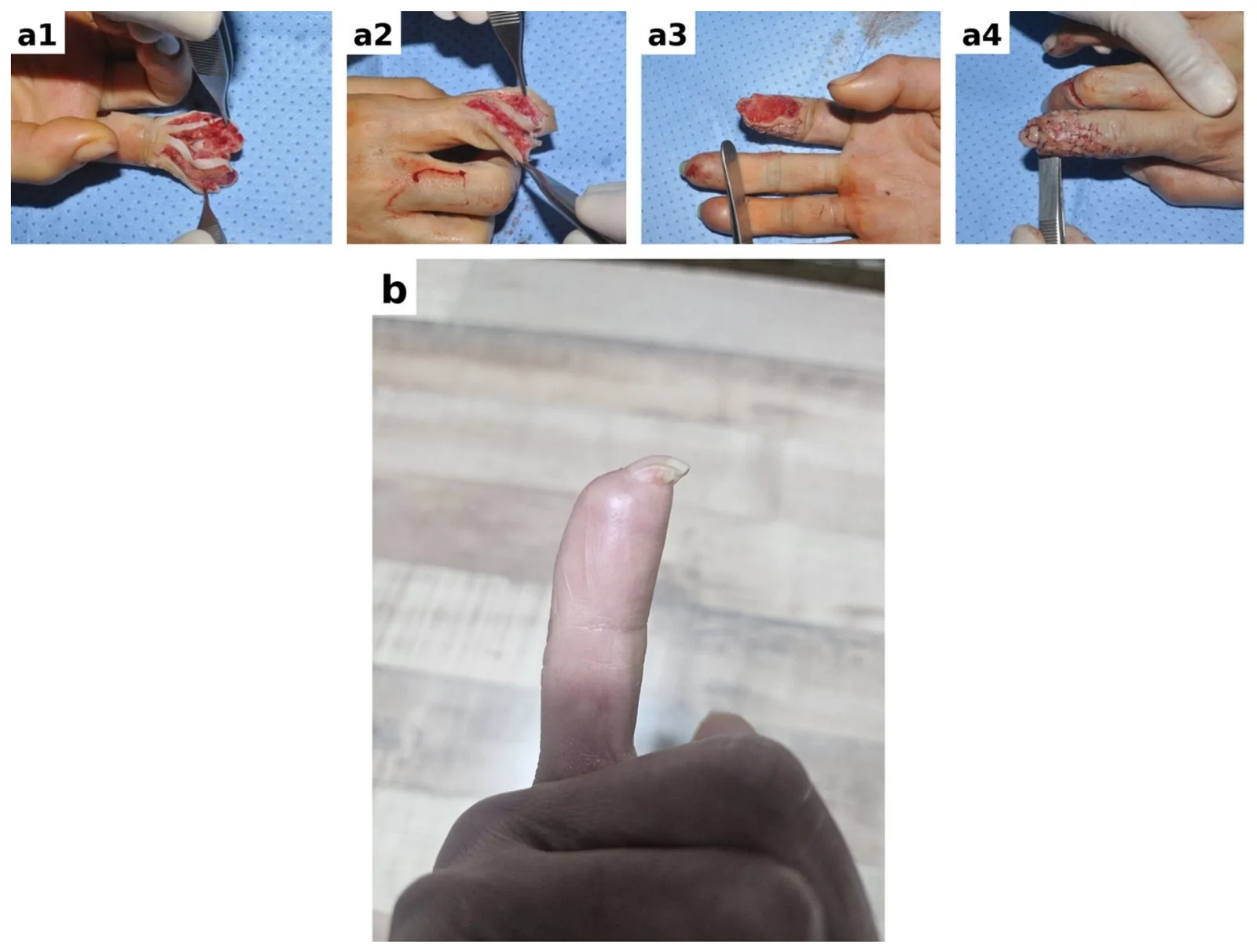
Allen type III index finger injury (digit no. 17) treated with primary repair of skin and nail bed followed by semi-occlusive dressing. (**a1**–**a4**) Injury day, before (**a1**,**a2**) and after repair (**a3**,**a4**). (**b**) Hook nail deformity at 32 months.

**Table 1:** Patient demographics and injury characteristics.

Characteristic	Value
Patients	15
Injured digits	17
Sex, male:female	11:4
Age (years), mean (range)	58 (39–79)
**Injured digit**	
Thumb	3
Index	3
Middle	1
Ring	5
Small	5
Multiple digit injury, patients	2
**Injury mechanism, patients**	
Crushing	5
Machinery-related	7
Cutting	2
Other	1
**Treatment**	
Dressing alone	7
Dressing after a surgical procedure	8

All injuries had bone exposure.

**Table 2:** Clinical outcomes.

Outcome	Result
Healing time (days), mean (range)	36 (21–60)
Complete wound healing	17/17
Follow-up (months), mean (range)	25 (12–63)
Additional surgery after initial treatment	0
Major complication (necrosis, non-healing)	0
**Nail deformity, digits**	
Total	4/17 (24%)
Hook nail	2
Dystrophic nail (severe 1, minor 1)	2
**Range of motion**	
Fingers, full recovery of total active motion	10/14
Fingers, total active motion (% of contralateral), mean (range)	95 (80–100)
Thumbs, interphalangeal joint motion (% of contralateral), mean (range)	80 (50–100)
**Sensory recovery**	
Two-point discrimination (mm), mean (range)	6 (3–10)
Semmes–Weinstein monofilament, median (range)	2.83 (2.83–4.31)
Subjective sensation (% of contralateral), mean (range)	84 (20–100)

Values are mean (range) unless otherwise indicated. Semmes–Weinstein results are ordinal; the index markings of the monofilament set are reported as labelled on the filaments, and all other values are rounded to whole numbers.

**Table 3:** Digit-level clinical data of the 17 injured digits.

Digit No.	Age/Sex	Digit	Defect Orientation	Germinal Matrix Involvement	Nail Bed Loss (%)	Nail Bed Repair	Mechanism	Surgery Before Dressing	Healing (Days)	Follow-Up (Months)	ROM (% Contra) ^a^	2PD (mm)	SW	Subjective Sensation (%)	Nail Deformity
1	67/M	Small	Transverse	No	10	No	Machinery	No	21	32	80	7	4.31	70	—
2	66/M	Ring	Transverse	No	30	Yes	Crushing	Yes	60	24	100	5	4.31	70	—
3	59/M	Ring	Transverse	No	90	No	Machinery	No	30	14	90	5	2.83	80	Dystrophic (minor)
4	39/M	Index	Transverse	No	80	Yes	Machinery	Yes	58	63	80	10	4.31	20	Hook
5	61/M	Middle	Lateral oblique	No	30	No	Machinery	No	30	13	100	5	2.83	90	—
6	53/F	Thumb ^b^	Transverse	No	60	No	Crushing	Yes	23	24	100	6	4.31	90	—
7	61/M	Ring	Dorso-oblique	No	60	No	Machinery	No	25	25	100	5	2.83	80	—
8	61/M	Thumb ^b^	Dorso-oblique	Yes	≥80	No	Machinery	No	25	25	90	5	2.83	50	Dystrophic (severe)
9	60/M	Ring	Transverse	No	70	Yes	Machinery	Yes	38	12	100	6	2.83	90	—
10	60/M	Small	Dorso-oblique	No	50	Yes	Machinery	Yes	38	12	100	6	3.61	90	—
11	39/M	Index	Not classifiable ^c^	Yes	≥80	Yes	Crushing	Yes	42	36	100	7	4.31	100	—
12	79/M	Small	Transverse	No	50	No	Crushing	Yes	21	13	100	6	3.61	100	—
13	72/F	Small	Dorso-oblique	Yes	50	No	Cutting	No	30	24	100	5	2.83	90	—
14	59/F	Small	Dorso-oblique	No	70	No	Other	No	25	30	100	6	2.83	100	—
15	65/M	Thumb ^b^	Not classifiable ^c^	No	40	No	Crushing	Yes	45	30	50	4	2.83	100	—
16	49/M	Ring	Transverse	No	90	No	Machinery	No	34	13	100	3	2.83	100	—
17	45/F	Index	Not classifiable ^c^	Yes	100	Yes	Cutting	Yes	60	32	80	7	3.61	100	Hook

2PD, static two-point discrimination; ROM, range of motion; SW, Semmes–Weinstein monofilament. Germinal matrix involvement denotes injury extending proximal to the sterile matrix; all remaining digits had injury confined to the sterile matrix. Nail bed loss is the proportion of the total nail bed area lost. Nail bed repair denotes primary repair performed at the initial procedure. ^a^ Total active motion for the fingers and interphalangeal joint motion for the thumbs, each expressed as a percentage of the contralateral digit. ^b^ Thumb; motion assessed as interphalangeal joint motion. ^c^ Irregular defect that could not be assigned to any of the listed orientations. Digit 17 sustained a mandoline-type injury with multiple parallel slice planes and a comminuted open fracture. Digits 7 and 8 belong to one patient, as do digits 9 and 10. Semmes–Weinstein results are ordinal; the index markings of the monofilament set are reported as labelled on the filaments, and all other values are rounded to whole numbers. Nail deformity developed in four digits of four patients: two hook nails and two dystrophic nails, one graded as severe and one as minor. All four occurred among the six digits with a nail bed loss of 80% or more, and none occurred among the 11 digits with a lesser loss (4/6 versus 0/11, *p* = 0.006). The mean nail bed loss was 88% in the digits that developed a deformity and 53% in those that did not (*p* = 0.01). By contrast, deformity was not related to germinal matrix involvement (2/4 versus 2/13, *p* = 0.22) or to whether the nail bed had been repaired (2/6 versus 2/11, *p* = 0.58).

**Table 4:** Comparison of digits treated with semi-occlusive dressing alone and after a surgical procedure.

	Dressing Alone (7 Patients, 8 Digits)	Dressing After Surgery (8 Patients, 9 Digits)	*p* ^e^
**Baseline characteristics**			
Age (years), patients	61 (49–72)	56 (39–79)	0.44
Sex (patients), male:female	5:2	6:2	>0.99
*Injury mechanism, digits*			**0.03**
Crushing	0	5	
Machinery-related	6	3	
Cutting	1	1	
Other	1	0	
*Defect orientation, digits*			0.10
Transverse	3	5	
Dorso-oblique	4	1	
Lateral oblique	1	0	
Not classifiable	0	3	
Germinal matrix involvement, digits	2/8	2/9	>0.99
Nail bed loss (%) ^f^	60 (10–90)	62 (30–100)	NA ^f^
Nail bed loss ≥ 80%, digits	3/8	3/9	>0.99
Nail bed repair, digits	0/8	6/9	NA ^d^
**Outcomes**			
Healing time (days)	28 (21–34)	43 (21–60)	**0.04 ^h^**
Complete wound healing, digits	8/8	9/9	–
Follow-up (months) ^g^	22 (13–32)	27 (12–63)	0.98
Additional surgery, digits	0/8	0/9	–
Nail deformity, digits	2/8	2/9	>0.99
Fingers, total active motion (% contralateral)	96 (80–100) ^a^	94 (80–100) ^a^	>0.99
Fingers, full recovery of total active motion	5/7	5/7	>0.99
Thumbs, interphalangeal joint motion (% contralateral)	90 ^b^	75 (50–100) ^c^	– ^i^
Two-point discrimination (mm)	5 (3–7)	6 (4–10)	0.08 ^h^
Semmes–Weinstein monofilament, median (range)	2.83 (2.83–4.31)	3.61 (2.83–4.31)	**0.02 ^h^**
Subjective sensation (% contralateral)	83 (50–100)	84 (20–100)	0.42

Values are mean (range) unless otherwise indicated. Bold *p*-values are below 0.05. ^a^ Seven fingers in each subgroup. ^b^ One thumb. ^c^ Two thumbs. ^d^ Nail bed repair is a component of surgical treatment and was possible only in digits that underwent a procedure; no between-group hypothesis test was performed (NA). ^e^ Two-sided tests, unadjusted for multiple comparisons: exact permutation Mann–Whitney U tests using midranks for continuous and ordinal variables, Fisher’s exact test for binary variables, and the Fisher–Freeman–Halton exact test for the overall distributions of injury mechanism and defect orientation. Age and sex were analyzed per patient; all other tested variables were analyzed per digit. Two patients each contributed two digits, so the digit-level tests do not account for within-patient clustering. All comparisons are exploratory, and the absence of a statistically significant difference does not establish equivalence. Dashes indicate invariant outcomes for which no test is informative. ^f^ Two nail bed loss entries were recorded as ≥80%; the descriptive summaries code these as 80%, and no continuous-variable test was performed because the exact percentages are unavailable. ^g^ Follow-up is reported per digit for consistency with the digit-level outcomes. ^h^ Sensitivity analysis with each patient contributing a single averaged value gave *p* = 0.08 for healing time, *p* = 0.14 for two-point discrimination and *p* = 0.02 for the Semmes–Weinstein value. ^i^ Thumb motion was described without testing because the subgroups contained only one and two thumbs. Semmes–Weinstein results are ordinal; the index markings of the monofilament set are reported as labelled on the filaments, and all other values are rounded to whole numbers.

## Data Availability

The data presented in this study are available on request from the corresponding author. The data are not publicly available because they contain information that could compromise the privacy of individual participants.
